# Multiple feature selection based on an optimization strategy for causal analysis of health data

**DOI:** 10.1007/s13755-024-00312-8

**Published:** 2024-11-12

**Authors:** Ruichen Cong, Ou Deng, Shoji Nishimura, Atsushi Ogihara, Qun Jin

**Affiliations:** 1 Graduate School of Human Sciences, Waseda University, 2-579-15 Mikajima, Tokorozawa, 359-1192 Saitama Japan; 2 Faculty of Human Sciences, Waseda University, 2-579-15 Mikajima, Tokorozawa, 359-1192 Saitama Japan

**Keywords:** Feature selection, Causal graphs, Health data analysis, Wearable device data

## Abstract

****Purpose**:**

Recent advancements in information technology and wearable devices have revolutionized healthcare through health data analysis. Identifying significant relationships in complex health data enhances healthcare and public health strategies. In health analytics, causal graphs are important for investigating the relationships among health features. However, they face challenges owing to the large number of features, complexity, and computational demands. Feature selection methods are useful for addressing these challenges. In this paper, we present a framework for multiple feature selection based on an optimization strategy for causal analysis of health data.

****Methods**:**

We select multiple health features based on an optimization strategy. First, we define a Weighted Total Score (WTS) index to assess the feature importance after the combination of different feature selection methods. To explore an optimal set of weights for each method, we design a multiple feature selection algorithm integrated with the greedy algorithm. The features are then ranked according to their WTS, enabling selection of the most important ones. After that, causal graphs are constructed based on the selected features, and the statistical significance of the paths is assessed. Furthermore, evaluation experiments are conducted on an experiment dataset collected for this study and an open dataset for diabetes.

****Results**:**

The results demonstrate that our approach outperforms baseline models by reducing the number of features while improving model performance. Moreover, the statistical significance of the relationships between features uncovered through causal graphs is validated for both datasets.

****Conclusion**:**

By using the proposed framework for multiple feature selection based on an optimization strategy for causal analysis, the number of features is reduced and the causal relationships are uncovered and validated.

## Introduction

The growth of information technology and the increase in wearable health data collection have created significant opportunities for health data analytics and the advancement of precision medicine [1, 2]. To enhance the quality of services in precision medicine, many studies have proposed personalized healthcare systems utilizing emerging technologies such as cloud computing, fog computing, Internet of Things, and mobile applications [3, 4]. These systems leverage health data for early disease detection and informed decision-making regarding individual health, aiming to improve quality of life. In addition, it is important to identify key patterns, trends, and relationships within complex health and medical data to further develop personalized healthcare and strengthen effective public health strategies. However, analyzing high-dimensional datasets from diverse wearable devices poses significant challenges, such as the curse of dimensionality and issues with interpretability. Feature selection is typically employed to enhance the performance of machine learning models by reducing the number of variables, streamlining the training process, and enhancing model comprehensibility. By determining the importance score for each feature, we select those with the highest scores to optimize model performance. Typically, features are ranked by their importance scores, and the top k most important features are selected. However, different feature selection methods can produce varying ranking results and feature combinations owing to differing criteria and algorithms, particularly when features interact strongly.

In contrast, the utilization and analysis of health data have become increasingly reliant on exploratory data analytics. Causal graphs, which are important for representing relationships between features, face challenges when there are numerous features and strong interactions in medical and health data. As the number of features increases, the complexity and interpretability of these causal graphs become more challenging, and analyzing and computing causal relationships demands more computational power. Furthermore, noise and redundant features in the dataset can obstruct accurate causality inference. Therefore, employing feature selection methods is necessary to determine key features and reduce the dataset’s dimensionality before creating causal graphs. It is important to determine which health indicators influence others and significantly impact an individual’s health, particularly in high-dimensional health data contexts.

In our previous work [5], we investigated how to identify important features from wearable device data to improve model accuracy through feature selection. We employed Extreme Gradient Boosting (XGBoost) for feature selection to reduce the number of features. The results indicated that using the top eight features for modeling resulted in an R-square score ($R^2$) of 0.365. Although this was an improvement over the results obtained using all features, the accuracy still leaves room for improvement. This suggests that the selected top eight features did not provide a sufficiently accurate representation of the target variables. Thereafter, we applied different feature selection methods and obtained rankings of feature importance. However, these methods often offer different perspectives, leading to differences in the importance rankings. In this study, we present a new framework for multiple feature selection based on an optimization strategy for causal analysis of health data. We aim to address the following research questions:

1. How can we combine different feature selection methods from multiple perspectives to determine the most important health features?
2. Is there a way to explore an optimal set of weights for combining multiple feature selection methods to obtain the index score for each health feature?
3. How can we uncover and validate the interrelationships among important health features?

The remainder of this paper is organized as follows: Sect. 2 reviews research related to health data analysis, feature selection, and causal discovery. In Sect. 3, we introduce our proposed framework for multiple feature selection based on an optimization strategy for causal analysis of health data, in addition to a detailed explanation of the multiple feature selection process. Section 4 describes the experiments conducted on two datasets and discusses the results. Finally, Sect. 5 concludes the paper and suggests potential future research directions.

## Related work

In this section, we review previous research on feature selection in the analysis of health data. After that, we present some studies related to machine learning and causal discovery in health data analysis that are relevant to our study.

### Feature selection in health data analysis

Feature selection plays an important role in health data analysis by improving model performance and interpretability. Numerous features can affect the precision and interpretability of models. Therefore, employing feature selection methods is essential when working with complex and diverse health data to reduce the number of features and identify important ones. For instance, Quaid and Jalal [6] introduced a novel feature selection method using genetic algorithms to analyze sensor data, simplify data complexity, and isolate key indicators. In the context of medical and health data, the presence of large-scale features results in a vast search space, leading to the wide-spread application of optimization algorithms for feature selection [7, 8]. Chen et al. [7] proposed a method using binary particle swarm optimization for feature selection aimed at improving the classification performance of healthcare data. Yang et al. [8] proposed a bi-directional particle swarm optimization framework that explores two directions. Their results showed an improvement in the performance of large-scale feature selection, yielding smaller feature subsets with higher accuracy. This approach provides a multi-directional strategy for reducing the number of features in large-scale data.

In addition, the results of ranking and the combinations of features generated by different feature selection methods vary significantly owing to the different criteria and algorithms employed. Pudjihartono et al. [9] provided a comprehensive overview of various feature selection methods, outlining their respective advantages and disadvantages as well as their applications in predicting based on medical data. These differences are particularly noticeable when features are highly interactive. Utilizing results from multiple models, known as ensemble learning, typically yields better results than relying on a single model. The concept can also be applied to feature selection. Zhang et al. [10] approached the feature selection challenge as an ensemble learning issue by combining weak classifiers with an optimized strong classifier. They presented a feature selection method based on proximity relational learning, where each feature was considered a binary classifier. However, this method has a high time complexity. Pardo et al. [11] employed different feature selection methods to train and identified important features based on a threshold, resulting in an applicable subset of features. Their findings demonstrated that this approach produced results that were at least as effective as, and often superior to, those achieved by any single feature selection method. However, the need for manual threshold adjustment undermines the objectivity of the feature selection process.

### Machine learning and causal discovery in health data analysis

This section examines the applications of machine learning and causal discovery in analyzing health data, highlighting significant studies and their findings. Many studies have employed statistical models or machine learning to analyze the relationships between health indicators [12, 13]. The availability of electronic medical records and data from wearable devices has also improved. Laffafchi et al. [14] proposed an approach for predicting Pulmonary Embolism diagnoses by analyzing electronic medical records, achieving an impressive F1 score of 0.96 through a two-step interconnected machine learning framework. Similarly, Thompson et al. [15] analyzed data from 6,907 individuals who self-reported having COVID-19, in addition to 1.1 million COVID-19 cases from electronic medical records. Their results demonstrated that several factors were associated with prolonged symptoms in both datasets. Wang et al. [16] proposed a diagnosis prediction model that represents electronic medical records as a patient-centered knowledge graph, employing spatial and temporal methods to predict patient conditions through multilabel classification. However, it is noteworthy that electronic health record data can include noise or subjective information from medical personnel and may pose accessibility challenges for individuals.

In contrast, wearable computing has emerged as an innovative approach to health analytics. Li et al. [17] proposed an end-to-end network for emotion recognition by leveraging the properties of electroencephalogram signals. Zhou et al. [18] analyzed how wearable devices affect the daily lives of elderly individuals and collected data on various metrics such as step count, sleep duration, blood pressure, heart rate, respiratory rate, fatigue, and mood. Their findings indicated significant changes in step count, blood pressure, and heart rate throughout the study. Interviews with older adults suggested that wearable devices could improve health and social capital. However, the pursuit of reliability in precision medicine has led to critical evaluations of current methodologies. Although these methods can predict diagnoses, they often fail to elucidate the causal relationships among health indicators. Consequently, causal inference has been proposed as a new approach to medical decision-making, helping to uncover useful relationships from real-world observational data and assess model reliability [19]. To support decision-making in health management and precision medicine, it is necessary to clear causal relationships. Prosperi et al. [20] used causal discovery algorithms to assess the validity of complex health indicators, whereas Shen et al. [21] presented a method for discovering causal structures specifically for electronic health records, demonstrating its application in type-2 diabetes mellitus. Similarly, Kotoku et al. [22] applied a direct linear non-gaussian acyclic model (DirectLiNGAM) to analyze health checkup data and identify causal relationships among health indicators, presenting results for specific subgroups.

This study integrates different feature selection methods and explores the optimal set of weights for these methods to identify important features that explain health status. Casual graphs are constructed using the selected features, and statistical tests are conducted to validate the paths.

## Multiple feature selection based on an optimization strategy for causal analysis

### Overall framework

**Fig. 1:**
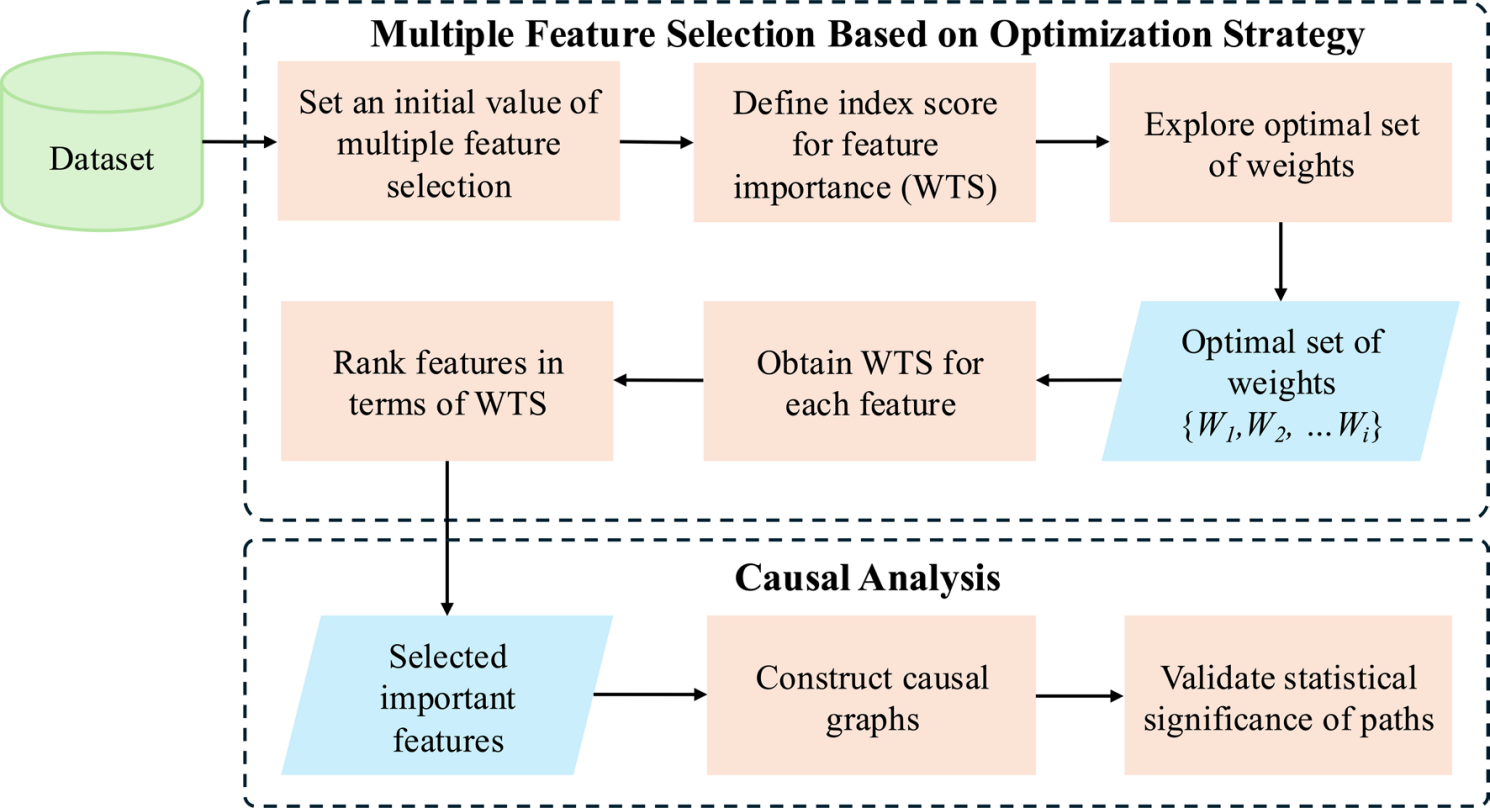
Overall Framework of Multiple Feature Selection Based on Optimization Strategy for Causal Analysis

In this study, we propose a new framework for multiple feature selection based on an optimization strategy for causal analysis of health data, as illustrated in Fig. 1. First, high-dimensional health data are collected from various sources, such as electronic health records, data from wearable devices, and biological sensing data. In the module of multiple feature selection based on optimization strategy, a combination of multiple feature selection methods is initiated. An index score, known as the Weighted Total Score (WTS), is defined to assess the importance of features identified by these combined methods. Following this, an optimal set of weights for the feature selection methods is explored, obtaining the calculation of the WTS for each feature. The features are then ranked based on their WTS, allowing for the selection of the most important ones. In the causal graph construction module, causal graphs on relationships between these features and the target outcomes are constructed. Finally, a statistical significance test is conducted to validate the pathways uncovered by the causal analysis. In summary, the framework aims to construct a new computational methodology on how to combine different feature selection methods based on an index score using an optimization strategy to determine important features while also uncovering and validating the relationships between these features and health outcomes through causal graphs. Further details on multiple feature selection based on the optimization strategy are described in Sect. 3.2.

### Defining weighted total score (WTS) for each feature

This section describes the definition of WTS for each feature. Owing to the diversity of feature selection methods available, relying on a single approach overlooks other important information. In this study, we define an index score, WTS, to assess the feature importance for the combination of different feature selection methods. We first define the feature importance scores for each method and use standard Min-Max normalization to adjust the values to a range of [0, 1], as shown in Eq. 1.

1 $$\begin{aligned} \tilde{F}_{ij} = \frac{F_{ij} - \min (F_{ij})}{\max (F_{ij}) - \min (F_{ij})} \end{aligned}$$

where *i* represents the *i*th method, *j* the *j*th feature, and $F_{ij}$ the importance score for the *j*th feature in the *i*th method.

Then, to obtain WTS, we use an exponential mapping function. The $WTS_j$ is defined in Eq. 2.

2 $$\begin{aligned} WTS_j = \sum _{i=1}^{n} W_i \cdot e^{\tilde{F}_{ij}} \end{aligned}$$

where *n* represents the number of feature selection methods. *e* is the base of the natural logarithm, and $W_i$ is the weight of the *i*th method, satisfying Eq. 3.

3 $$\begin{aligned} \sum _{i=1}^{n} W_i = 1 \end{aligned}$$

Equation 2 has two important components: the exponential function mapping and the weight for each method. The use of an exponential function enhances the differences between the feature importance scores. Minor differences are magnified after the exponential transformation, highlighting the more important features. Additionally, it provides nonlinear scaling, which is useful when the importance scores vary widely, ensuring that the significant features are more prominent. Finally, it maps all real numbers to positive values while maintaining a consistent and interpretable scale for feature importance.

By combining multiple feature selection methods and assigning them different weights, the potential biases of single methods were minimized, leading to a more balanced and fair feature selection process. This enables the model to adapt to different datasets and feature selection scenarios by adjusting the weights based on performance and relevance. Finally, the features are ranked based on WTS, and the top *k* features are selected, which can be represented as Eq. 4.

4 $$\begin{aligned} \text {Selected Features} = \{X_j \mid WTS_j \text { is among the top } k\} \end{aligned}$$

where *k* represents the number of features and $X_j$ represents the set of selected features.

### Exploring optimal set of weights for each method

This section provides details on how to explore an optimal set of weights ($W_i$) for each method. In this study, we use a greedy algorithm to explore and determine the optimal set of weights. It requires inputting feature scores from each feature selection method (*F*), feature variables (*X*), target variable (*y*), weight step size ($\delta$), maximum iterations (*T*), and number of features to be selected (*k*).

The process starts with the initialization of each selection method. The weight for each method is iterated according to the step size. At each iteration, WTS is obtained, features are ranked, and top k features are selected by the minimum mean squared error (MSE) and then by the maximal *R*^2^ if the values of MSE are the same. If better metrics are found, the current weights and metrics are updated accordingly. To enhance computational efficiency, a dictionary is used to store the evaluation results for each feature subset. Before each evaluation, it checks if a feature subset is already in the dictionary. If it is, the computation is skipped, and the stored result is used. This process continues until no further improvements can be made or the maximum number of iterations is reached. Then, the optimal set of weights ($W^*$), number of selected features ($k^*$), and best metrics ($MSE^*$ and $R^2*$) are provided as output. The details of this algorithm are shown in Algorithm 1.

**Algorithm 1:**
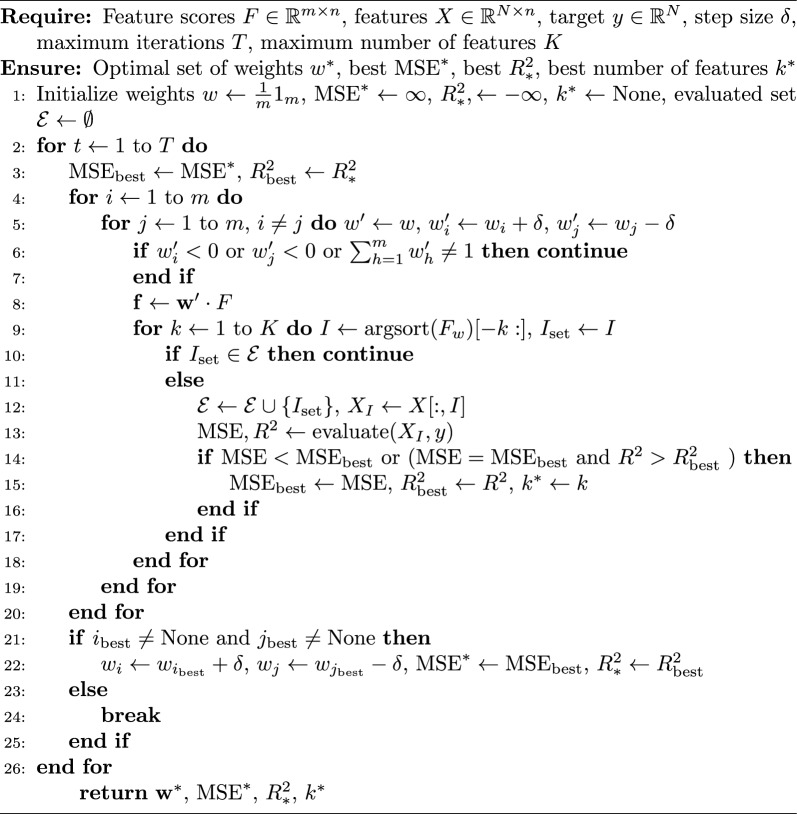
Multiple feature selection for exploring optimal set of weights

## Experiment and discussion

### Overview of datasets

#### WD-health dataset

From December 5, 2022, to January 26, 2023, this study recruited 22 undergraduate and graduate students, comprising 11 men and 11 women. The experimental procedures were approved by the Ethics Review Committee on Research with Human Subjects of Waseda University, Japan (Approval Nos. 2018-092 and 2022-436) and were conducted in accordance with the Declaration of Helsinki for experiments involving humans. All participants provided written informed consent. The data collection was divided into two parts. The first part was conducted in a laboratory at Waseda University, where Traditional Chinese Medicine (TCM) staff conducted weekly observations, questioning, and pulse data collection to assess TCM health scores (TCMHS) on a scale from 1 to 10 based on TCM-related data. This approach merges predictive medicine with the TCM principle of "Preventive Treatment of Diseases," providing a new perspective on identifying health risks through health data collected from various sensors and devices [23].

The second part was conducted in the participants’ daily lives. Participants were asked to wear wearable devices to monitor their daily activities and health indicators, such as heart rate, step count, and sleep quality. The wearable device, Huawei Band 7, which is a smart bracelet worn on the wrist, records 16 health indicators, as presented in Table 1. Participants were instructed to wear the device for 24 h a day, except while showering or charging. The device continuously transmitted health data via Bluetooth to a paired smartphone, which stored the data in the cloud.

**Table 1:** Feature description of WD-health dataset

Category	Feature	Description
Emotional	StressS	Stress score
Bio	RHR	Resting heart rate
	MinBO	Minimum blood oxygen
	MaxBO	Maximum blood oxygen
Sleep	SleepS	Sleep score
	DeepSC	Deep sleep continuity
	WUC	Wake up counts
	BreathQ	Breathing quality
	TSD	Total sleep duration
	TDSD	Total deep sleep duration
	TLSD	Total light sleep duration
	TRSD	Total rapid eye movement sleep duration
Activity	ACC	Activity calories consumption
	StepN	Step number
	StepD	Step distance
	MHIET	Moderate to high-intensity exercise time

#### Open dataset for diabetes

We also used an open dataset comprising data from 442 diabetes patients [24]. The dataset included variables such as age, sex, body mass index (BMI), average blood pressure (ABP), total cholesterol (TC), low-density lipoprotein (LDL), high-density lipoprotein (HDL), total cholesterol/HDL (TCH), logarithm of serum triglyceride level (LTG), blood glucose value (GLU), and the target variable, which indicated disease progression one year after baseline (Target).

### Determining important health features

#### Selecting features based on WTS

To integrate multiple feature selection methods, we used Pearson correlation (Pearson), distance correlation (Distance), Lasso regression (Lasso), random forest (RF-MDA), and stability selection (Stable). Each method was applied to generate rankings of feature importance. Furthermore, we assigned equal weights (0.2) to each method (Average Weight) to obtain the average weight scores and obtained rankings based on this average weight. Then, we explored the optimal set of weights to obtain WTS by using our proposed approach. The top k features from each method were selected to construct models for predicting outcomes. The optimal number of features, k, was identified by comparing the MSE and $R^2$ scores. In addition, we evaluated the root mean square error (RMSE), mean absolute error (MAE), and explained variance (EV) to ensure that the selected feature sets were consistent across different evaluation metrics.

We conducted the evaluation experiments using two datasets. First, with the WD-health dataset, our approach resulted in an optimal k value of six, with an MSE of 0.008, RMSE of 0.090, MAE of 0.046, $R^2$ of 0.536, and EV of 0.539. These results are summarized in Table 2. We reduced the number of selected features, and the model’s metrics outperformed those of single methods and the average weight method. The WTS values for the WD-health dataset are listed in Table 3, and the selected features were used for constructing causal graphs.

For the open diabetes dataset, our approach resulted in an optimal k value of seven, with an MSE of 0.021, RMSE of 0.145, MAE of 0.116, $R^2$ of 0.647, and EV of 0.648, as summarized in Table 4. The WTS rankings for each variable are presented in Table 5, highlighting the seven features with the highest WTS: LTG, BMI, TC, ABP, LDL, sex, and GLU. These seven most important features were used in causal graphs owing to their important impact on the target variable.

**Table 2:** Comparison of proposed approach and baselines on WD-health dataset

	Optimal k	MSE	RMSE	MAE	R^2^	EV
Pearson	13	0.009	0.095	0.051	0.487	0.489
Distance	15	0.009	0.095	0.052	0.479	0.483
RF-MDA	9	0.009	0.095	0.051	0.496	0.496
Lasso	15	0.009	0.095	0.050	0.511	0.513
Stable	8	0.009	0.095	0.049	0.504	0.505
Average weight	13	0.009	0.095	0.051	0.488	0.489
Our approach	**6**	**0.008**	**0.090**	**0.046**	**0.536**	**0.539**

Bold values indicate the best values for the indicator in the different methods

Underlined values indicate the next best value for the indicator in the different methods

**Table 3:** Weighted total score (WTS) for WD-health dataset

Feature	WTS	Feature	WTS
WUC	2.422	MHIET	1.074
StressS	2.401	SleepS	1.062
StepN	2.133	MinBO	1.060
StepD	2.011	TLSD	1.057
BreathQ	1.198	TRSD	1.051
TSD	1.165	MaxBO	1.047
ACC	1.146	DeepSC	1.037
RHR	1.077	TDSD	1.008

**Table 4:** Comparison of proposed approach and baselines on open dataset for diabetes

	Optimal k	MSE	RMSE	MAE	R^2^	EV
Pearson	10	0.024	0.155	0.122	0.594	0.597
Distance	10	0.024	0.155	0.122	0.594	0.597
RF-MDA	**5**	0.023	0.152	0.123	0.617	0.619
Lasso	7	0.024	0.155	0.122	0.604	0.606
Stable	8	0.023	0.152	0.122	0.613	0.607
Average weight	10	0.024	0.155	0.122	0.594	0.597
Our approach	7	**0.021**	**0.145**	**0.116**	**0.647**	**0.648**

Bold values indicate the best values for the indicator in the different methods

Underlined values indicate the next best value for the indicator in the different methods

**Table 5:** Weighted total score (WTS) for open dataset for diabetes

Feature	WTS	Feature	WTS
LTG	2.655	Sex	1.127
BMI	2.445	GLU	1.109
TC	1.453	TCH	1.100
ABP	1.345	Age	1.048
LDL	1.241	HDL	1.047

#### Investigating multicollinearity on selected features

**Fig. 2:**
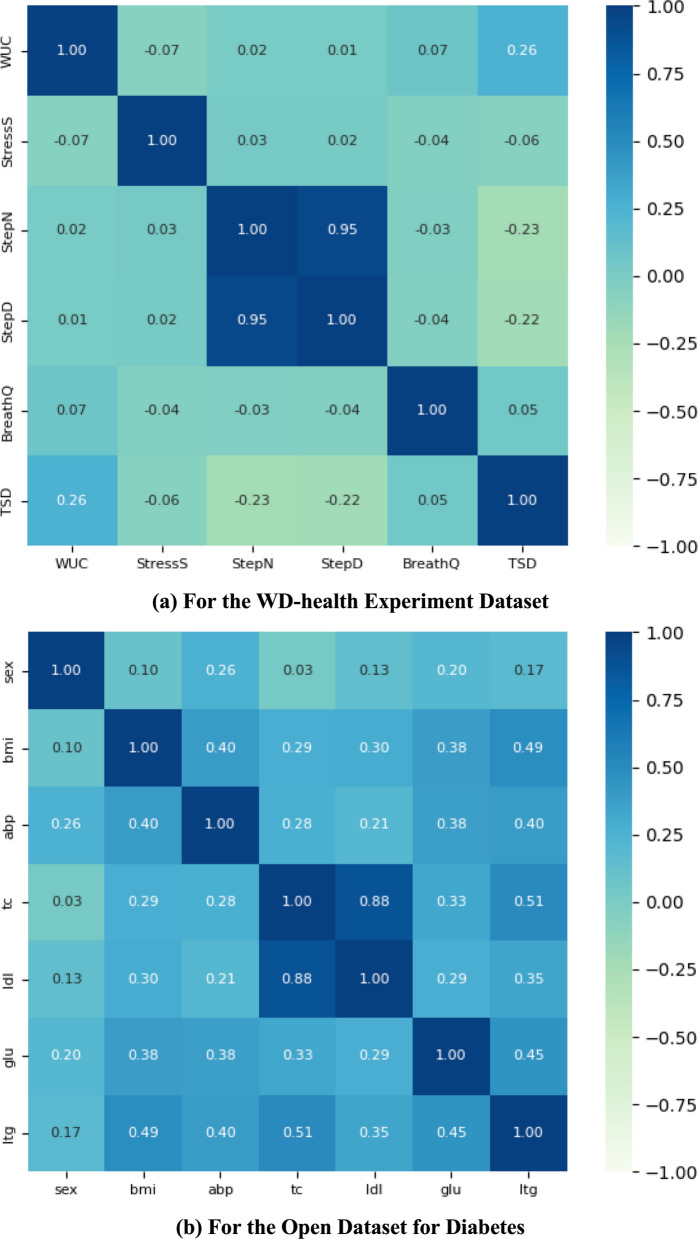
Correlation heatmaps for WD-health experiment dataset and open dataset for diabetes

To prevent multicollinearity, we conducted a correlation analysis on the selected features. Figure 2(a) shows a heat map of the Spearman correlation coefficients among the features from the WD-health dataset. For instance, Step Number (StepN) and Step Distance (StepD) showed strong positive correlations (r = 0.95), indicating potential multicollinearity. We assessed multicollinearity using variance inflation factors (VIFs), which revealed that StepD and StepN had VIFs of 15.34 and 15.25, respectively, indicating significant multicollinearity. After removing StepD, the VIFs for the remaining features dropped below 10. Therefore, the selected features are Wake Up Counts (WUC), Stress Score (StressS), StepN, Breathing Quality (BreathQ), and Total Sleep Duration (TSD).

The correlation heatmap between the selected features from the open diabetes dataset is shown in Fig. 2(b). A positive correlation exists between any pair of indicators, indicating that key factors influencing the progression of diabetes tend to increase together. Among them, TC and LDL levels showed a strong positive correlation (r = 0.88), suggesting potential multicollinearity between these two features. To further analyze this, we investigated the VIF for each indicator. The VIF values for the seven selected indicators-LTG, BMI, TC, ABP, LDL, Sex, and GLU-are all below the acceptable threshold of 10.

**Fig. 3:**
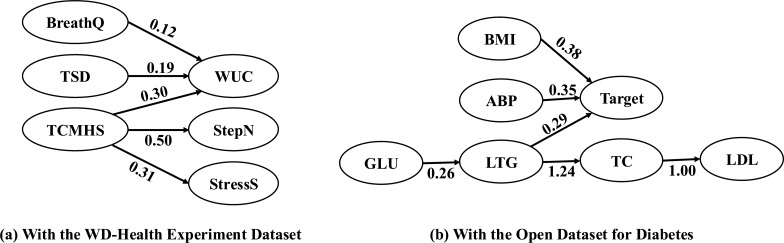
Causal graphs with WD-health experiment dataset and open dataset for diabetes

### Construction and validation of causal graphs

#### Construction of causal graphs

In this study, we employed DirectLiNGAM [25, 26], a causal discovery algorithm, to investigate the causal paths between selected health features and health outcomes. DirectLiNGAM excels in exploring causal relationships within non-Gaussian datasets. It determines the causal order of variables through independent tests, eliminating the need for prior knowledge. This capability is particularly pertinent for health data, which frequently deviate from Gaussian distributions. It is based on a clear mathematical model that enhances interpretability in the healthcare and medical fields. It effectively handles confounders and is robust to outliers, addressing the complexity inherent in health data relationships.

We constructed Directed Acyclic Graphs (DAGs) that estimate the causal structures linking important health features and outcomes. DAG consists of vertices (nodes) connected by directed edges without cycles, representing the relationships and dependencies between variables. In this graph, the nodes represent variables, arrows indicate the direction of influence, and coefficients along the paths quantify the strength of these relationships.

#### Validation of causal relationships

The fitting and analysis of Structural Equation Modeling (SEM) [27] models play an important role in the graph model. To validate the reliability of the causal graph, we conducted a hypothesis test using SEM. This method evaluates the consistency of a hypothesis with the collected data by modeling the relationships between variables. By comparing model predictions with real data, we could verify the validity of the hypotheses. The models were fitted and analyzed using the Python semopy library [28] to validate and evaluate the statistical significance of the paths.

Figure 3 (a) depicts the five selected health features within the causal graph of the TCMHS. In this graph, TCMHS directly affects WUC, StepN, and StressS, with path coefficients of 0.30, 0.50, and 0.31, respectively. Additionally, Fig. 3 (a) shows the complex interactions among the health features. Both BreathQ and TSD directly affect WUC, with path coefficients of 0.12 and 0.19, respectively, suggesting that WUC serves as a mediator variable influenced by multiple factors. The results of the statistical tests are presented in Table 6, where all the analyzed paths showed significance with p < 0.05 or p < 0.001.

Figure 3 (b) depicts the seven selected features in the causal graph of the target. In this graph, the target is directly affected by BMI, ABP, and LTG, with path coefficients of 0.38, 0.35, and 0.29, respectively. Additionally, Fig. 3 (b) shows the complex interactions among the independent indicators. The path from GLU to LTG, then to TC, and finally to LDL shows direct effects with path coefficients of 0.26, 1.24, and 1.00, respectively. The results of the statistical tests are summarized in Table 7, where all paths showed significance with p < 0.001.

**Table 6:** Results of hypothesis test on WD-health dataset

Path			Estimate	Std. Err	z-value	p-value
TCMHS	$\rightarrow$	WUC	0.370	0.031	11.755	<0.001
TSD	$\rightarrow$	WUC	0.350	0.039	9.025	<0.001
BreathQ	$\rightarrow$	WUC	0.075	0.026	2.833	<0.05
TCMHS	$\rightarrow$	StressS	0.397	0.032	12.358	<0.001
TCMHS	$\rightarrow$	StepN	0.488	0.027	17.954	<0.001

*Note. Results in the table are considered statistically significant at the p* < *0.05 level*

**Table 7:** Results of hypothesis test on open dataset for diabetes

Path			Estimate	Std. Err	z-value	p-value
GLU	$\rightarrow$	LTG	0.489	0.044	11.037	<0.001
LTG	$\rightarrow$	TC	0.477	0.038	12.647	<0.001
LTG	$\rightarrow$	Target	0.440	0.046	9.563	<0.001
ABP	$\rightarrow$	Target	0.200	0.046	4.305	<0.001
BMI	$\rightarrow$	Target	0.490	0.049	9.903	<0.001
TC	$\rightarrow$	LDL	0.801	0.019	42.577	<0.001

*Note. Results in the table are considered statistically significant at the p* < *0.05 level*

### Discussion

Tables 2 and 4 present comparisons of our proposed approach against the baselines for the two datasets. Our approach outperformed the baselines in most metrics while determining fewer features. Specifically, the model for the WD-health dataset accounted for 53.6% of the variation in the target variable, with the remaining 46.4% attributed to other factors. Additionally, the model for the open diabetes dataset explained 64.7% of the variation in its target variables. Although the number of selected features (k) in our approach for the open diabetes dataset was not the lowest, it was the second best. Figures 3 (a) and (b) show the causal relationships within the two datasets. Figure 3 (a) highlights the connections between mental health, physical activity, sleep quality, and overall health, whereas Fig. 3 (b) highlights the importance of BMI and ABP in daily health management and suggests that LTG serves as a key indicator that interacts with other indicators.

In our previous study [29], we proposed a framework for estimating latent risk factors and health abnormalities using a domain model, which requires expert knowledge for its construction. In [30], we proposed using causal discovery algorithms to construct causal graph models as alternatives to expert-based domain models. However, selecting important features before constructing causal graphs is necessary. In this study, we proposed a combination of multiple feature selection methods to determine these important features. By using multiple feature selections, we can estimate latent risk factors and health issues without relying on domain expertise.

Our proposed framework for multiple feature selection based on an optimization strategy for causal analysis of health data has potential applications in personalized healthcare, where wearable devices are employed to continuously monitor daily health indicators such as heart rate, sleep quality, and blood oxygen. By identifying the important features and uncovering causal relationships between health indicators, individuals can manage their health in an objective manner and improve their lifestyles and behaviors. Furthermore, the early warning signs of lifestyle-related diseases such as diabetes and cardiovascular diseases can be detected. Moreover, it can be integrated within mobile healthcare applications as a personal AI health assistant, offering individuals personalized health advice based on their data through multimodal data fusion, including text, audio, or images.

However, this study has limitations, including its sensitivity to data quality and the need for large and diverse sample sizes to ensure robustness.

## Conclusion

In this study, we proposed a framework for multiple feature selection based on an optimization strategy for causal analysis of health data. The main contributions of this study are summarized as follows. (1) We proposed a new approach to multiple feature selection using an optimization strategy based on a new index score called WTS for each health feature. (2) We explored and determined the optimal set of weights for different selection methods to obtain the WTS of each feature, ranking the features by their WTS and selecting the most important features. (3) We further constructed causal graphs to uncover the relationships between the selected features and tested the statistical significance of the paths.

Furthermore, we conducted evaluation experiments on two datasets: the WD-health dataset collected for this study and an open dataset for diabetes. The results demonstrated that our approach outperformed the baselines by reducing the number of features while improving model performance. In addition, we uncovered the relationships between features using causal graphs and validated their statistical significance for both datasets.

For our future work, we will integrate our proposed approach into application services to enable and facilitate personolized precision healthcare in daily life.

## Data Availability

The WD-health dataset generated and analyzed in this study is not publicly available due to their containing information that could compromise the privacy of experiment participants, but is available from the corresponding author on reasonable request. The open dataset for diabetes is available from https://www4.stat.ncsu.edu/~boos/var.select/diabetes.html
